# Potential of feedback during objective structured clinical examination to evoke an emotional response in medical students in Canada

**DOI:** 10.3352/jeehp.2020.17.5

**Published:** 2020-02-18

**Authors:** Dalia Limor Karol, Debra Pugh

**Affiliations:** 1 University of Ottawa Faculty of Medicine, Ottawa, ON, Canada; 2 Department of Medicine, University of Ottawa, Ottawa, ON, Canada; 3 Medical Council of Canada, Ottawa, ON, Canada; Hallym University, Korea

**Keywords:** Canada, Embarrassment, Feedback, Medical students, Physical examination

## Abstract

Feedback has been shown to be an important driver for learning. However, many factors, such as the emotional reactions feedback evokes, may impact its effect. This study aimed to explore medical students’ perspectives on the verbal feedback they receive during an objective structured clinical examination (OSCE); their emotional reaction to this; and its impact on their subsequent performance. To do this, medical students enrolled at 4 Canadian medical schools were invited to complete a web-based survey regarding their experiences. One hundred and fifty-eight participants completed the survey. Twenty-nine percent of respondents asserted that they had experienced emotional reactions to verbal feedback received in an OSCE setting. The most common emotional responses reported were embarrassment and anxiousness. Some students (n=20) reported that the feedback they received negatively impacted subsequent OSCE performance. This study demonstrates that feedback provided during an OSCE can evoke an emotional response in students and potentially impact subsequent performance.

## Background/rationale

Objective structured clinical examinations (OSCEs) are performance-based examinations that are typically used to assess clinical (e.g., physical examination and history taking) and communication skills. During an OSCE, examinees rotate through a number of stations in which they may be asked to interact with a standardized participant (SP) while being directly observed by an examiner. OSCEs are a frequently-used tool of assessment in health professions education.

In addition to being a useful method to assess learning, OSCEs have also been shown to drive learning [1]. One important way that OSCEs help promote learning is through verbal feedback, which is often provided by examiners at the end of each station during formative OSCEs. This feedback can thus be used by trainees to self-regulate their learning. However, the learners’ confidence, comfort level and experience have been shown to influence the way in which they receive feedback [2]. So, although verbal feedback is often provided during formative OSCEs, there are differences in how trainees may internalize and use this feedback.

Although educators may view formative assessment as an opportunity to guide learning by providing feedback, learners may view all examinations as hurdles that must be overcome, rather than as genuine opportunities to learn [3]. Perhaps this is because there is an inherent social judgment associated with feedback that can lead to a negative emotional reaction. This may be particularly true when the feedback provided is perceived as critical or in some way threatening to one’s sense of self. In such cases, examinees may either discount it (because it is not congruent with their self-assessments) or avoid seeking out future feedback [2]. If the feedback received evokes a strong emotional response, it is unclear how this may impact examinees’ subsequent performance.

## Objectives

In light of the variability of feedback given during an OSCE, and feedback’s inherent ‘sting’ [3], this study aimed to explore medical students’ perspectives on the feedback they receive during an OSCE; their emotional reaction to this; and the subsequent impact on their performance on other stations.

## Ethics statement

We received ethical approval from University of Ottawa Faculty of Medicine (REB #20180668-01H). Informed consent was obtained from subjects.

## Study design

This is a survey result-based descriptive study.

## Settings and participants

We reached out to representatives from each of the anglophone medical schools in Canada (n=13) to invite them to distribute a web-based survey from November 2018 to June 2019 through Survey Monkey. Four schools agreed to participate (University of Ottawa, University of Toronto, Queen’s University, and Western University), meaning the survey was sent to approximately 2,000 students. The survey included 13 questions in both constructed- and selected-response formats.

## Statistical methods

Descriptive statistics were used to analyze the answers to selected-response questions. Comments were analyzed using content analysis.

## Descriptive data

**Fig. 1.**
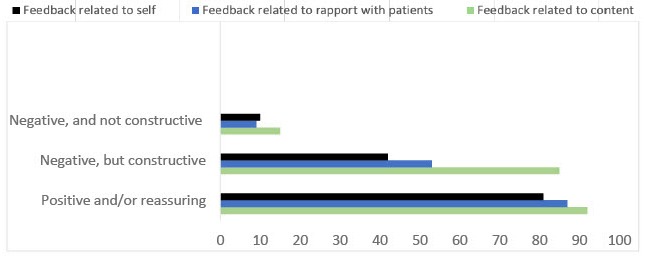
Type of feedback received by students during an objective structured clinical examination.

One hundred and fifty-eight students completed the survey. Respondents were relatively evenly distributed among 2nd, 3rd, and 4th year (60, 51, and 43, respectively). Additionally, 4 students were in their first year of study. All respondents had participated in at least one OSCE, with 75 (47.5%) having participated in more than 3 OSCEs. The 113 (71.5%) of respondents reported receiving verbal feedback during an OSCE. Of those who received verbal feedback, all reported receiving feedback from a physician examiner, with 60.2% having also received feedback from an SP and 6.1% from a fellow student. One hundred and fifty one respondents (95.6%) reported receiving positive and/or reassuring feedback, 140 (88.6%) received negative, but constructive feedback, and 25 (15.8%) received negative, non-constructive feedback (Fig. 1). Feedback was most frequently related to content, followed by feedback related to the candidates’ ability to establish rapport.

**Fig. 2.**
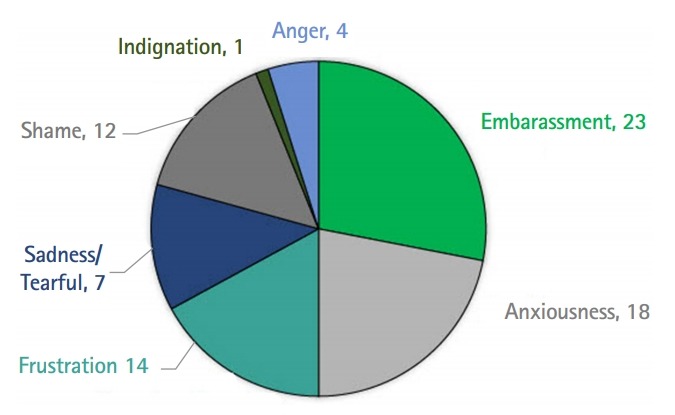
Emotions experienced by students in response to feedback received during an objective structured clinical examination that they deemed was too harsh, rude, unfair, or inappropriate.

Twenty nine percent of participants reported having an emotional reaction to negative verbal or nonverbal feedback received during an OSCE. The most common emotions experienced were embarrassment, anxiousness, and frustration (Fig. 2).

When respondents were asked to provide examples of verbal and/or nonverbal feedback that led to their emotional reaction, many identified non-verbal cues, such as examiners that rolled their eyes, loudly sighed, and used a ‘harsh’ or ‘patronizing’ tone while giving feedback. Specific quotes were given of examiners that gave negative and non-constructive feedback or used sarcasm in their giving of feedback.

R1: “Do you even know what you are saying?”
R2: “Eye rolling ‘looks like everyone in the city is going to get tetanus’—referring to the fact that several students forgot to ask about tetanus status at this station.”

Some respondents reported that the negative feedback they received was as a result of the examiner not hearing or mishearing them

R3: “Examiner said I ignored patient comfort and that when the patient had said she was in pain I had just answered with “OK”. I had actually asked the patient if she was OK to continue. Patient defended me to the examiner. Was frustrated because he accused me of doing something I had not done and it felt like he had not even been paying attention to my performance.”

Other respondents had emotional reactions to feedback, for example when they felt as though the examiner was being too picky about their choice of words

R4: “Pointing out things I had done wrong, especially very picky details (for example I used the term ‘authorities’ rather than ‘police’ and was criticized heavily for this).”

Comments about a lack of empathy arose frequently as evoking emotional reactions during the OSCE feedback process.

R5: “At the end of the station, the physician preceptor told me that I am not very empathetic, not attentive, and that I missed a large piece of that station. I was very offended and I almost burst into tears because I definitely consider myself an empathetic, attentive, and kind person. This interaction really affected me.”

Respondents reported that the feedback they received impacted their performance on subsequent OSCE stations in a negative way by increasing their anxiety and/or decreasing their self-confidence (n=20). Other respondents reported that they were able to suppress the emotional reaction and thus it had no effect on subsequent stations (n=6). One respondent reported that negative feedback led to improved performance and stated that “it made me better”. Several respondents (n=8) reported that the feedback influenced their study behaviors in both positive and negative ways.

R6: “I made sure to prepare extensively for future follow-up questions for future OSCEs so that I would not feel embarrassed again.”
R7: I think it made me more nervous than necessary for future OSCEs which reduced my studying efficacy.
R8: “Studied harder to screw over these type of -------”

## Interpretation

This study sought to explore medical students’ perspectives on the feedback they receive in OSCEs. Although much of this feedback is perceived as constructive, a significant number of comments are perceived as negative and non-constructive. In many cases, this led students to experience negative emotions. Although some students appear to be able to use this feedback as an impetus for learning, for others it was detrimental.

## Comparison with previous studies

This is concordant with the literature, as some studies report that when feedback is given during an OSCE it is viewed by students as helpful [4]. However, other studies demonstrate that feedback can elicit strong emotional responses and affect students’ self-esteem [5]. Negative emotional reactions can be both detrimental and/or beneficial to performance [6].

## Limitations

This study is not without limitations. There may be systematic differences in people who respond to surveys compared to those who do not [7]. Since there was no incentive offered for this study, there is a possible selection bias towards students who had received memorable feedback during their OSCEs. Another limitation of this study is that the survey tool was not validated and a reliability test was not done.

## Conclusion

Ultimately, the majority of students did not report having negative reactions to feedback in an OSCE setting. However, negative and non-constructive feedback given during an OSCE has the potential to evoke an emotional response in some students. The emotional responses, most commonly embarrassment and anxiousness, have the potential to impact subsequent OSCE performance. Further training can be provided to examiners on how to provide negative and constructive feedback. Additionally, providing students with education about the purpose of feedback and how it can be used to guide their learning may help them to approach the way they receive feedback differently. Training students in this way, may help them develop resiliency to feedback. Further research can use qualitative interviews to explore differences between students who experience negative emotional responses, and those who do not.

## Supplementary materials

Data files are available from https://doi.org/10.7910/DVN/GHNWKA

Supplement 1. Survey questionnaire to explore the feedback received by medical students during OSCEs in the University of Ottawa Faculty of Medicine from November 2018 to June 2019.

Supplement 2. Audio recording of the abstract.
