# Supplementary material for: Potential of feedback during objective structured clinical examination to evoke an emotional response in medical students in Canada
Source: J Educ Eval Health Prof. 2020 Feb 18;17:5. doi: 10.3352/jeehp.2020.17.5 (PMC7136617; doi:10.3352/jeehp.2020.17.5)
Supplement: Supplementary file 1 [file jeehp-17-05-dataset1.pdf]

Q1 Participation is completely voluntary. If you decide to participate, you are free to discontinue responding to the survey at any time, without penalty or loss. Survey responses are anonymous and no names or contact information will be collected. Therefore, once the completed survey has been submitted, withdrawal will not be possible

Answered: 158 Skipped: 0

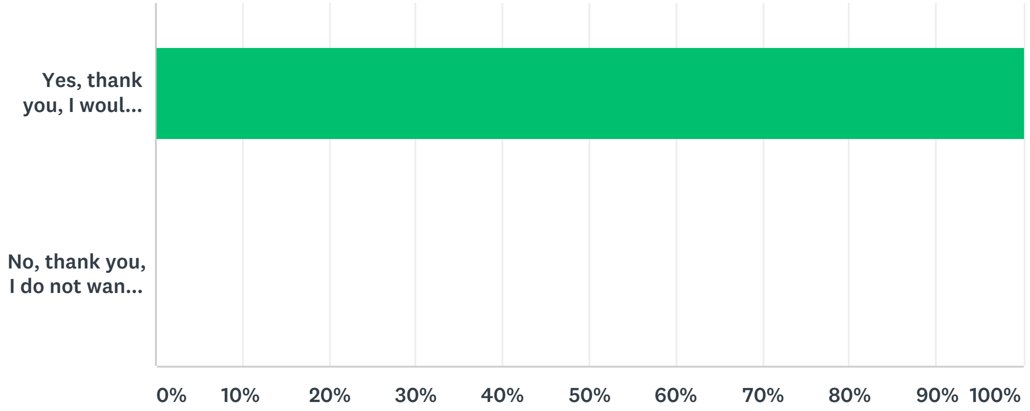

ANSWER CHOICES	RESPONSES	
Yes, thank you, I would like to start the survey	100.00%	158
No, thank you, I do not want to participate	0.00%	0
TOTAL		158

Q2 1. In what year of medical school are you currently enrolled?

Answered: 151 Skipped: 7

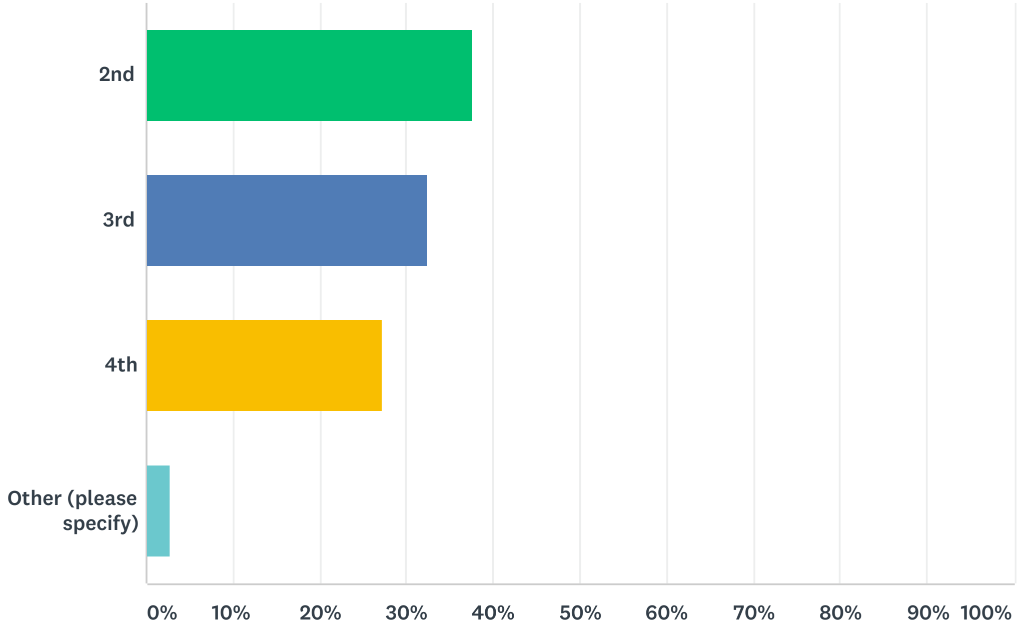

ANSWER CHOICES	RESPONSES	
2nd	37.75%	57
3rd	32.45%	49
4th	27.15%	41
Other (please specify)	2.65%	4
TOTAL		151

Q3 2. How many OSCEs have you participated in as a medical student?

Answered: 151 Skipped: 7

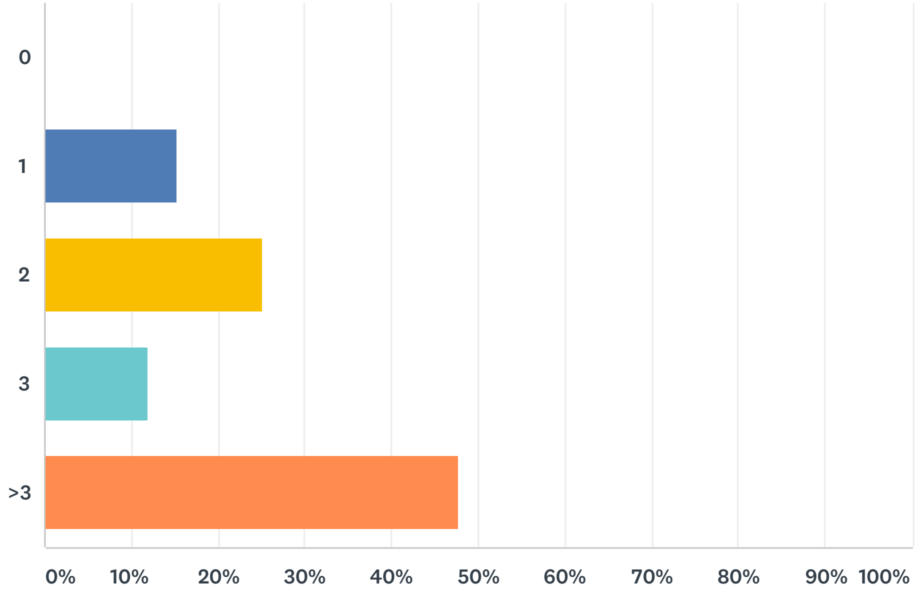

ANSWER CHOICES	RESPONSES	
0	0.00%	0
1	15.23%	23
2	25.17%	38
3	11.92%	18
>3	47.68%	72
TOTAL		151

Q4 3. During your OSCEs, have you ever received verbal feedback from an examiner?

Answered: 150 Skipped: 8

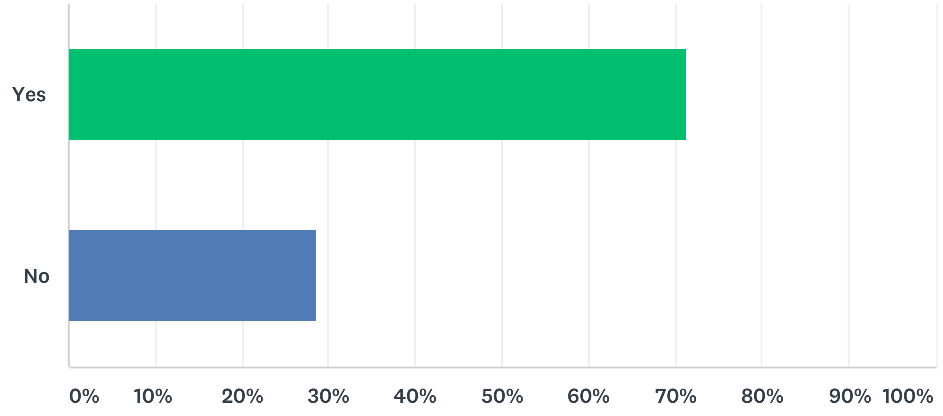

ANSWER CHOICES	RESPONSES	
Yes	71.33%	107
No	28.67%	43
TOTAL		150

Q5 4. Who provided the verbal feedback? Select as many as applicable

Answered: 98 Skipped: 60

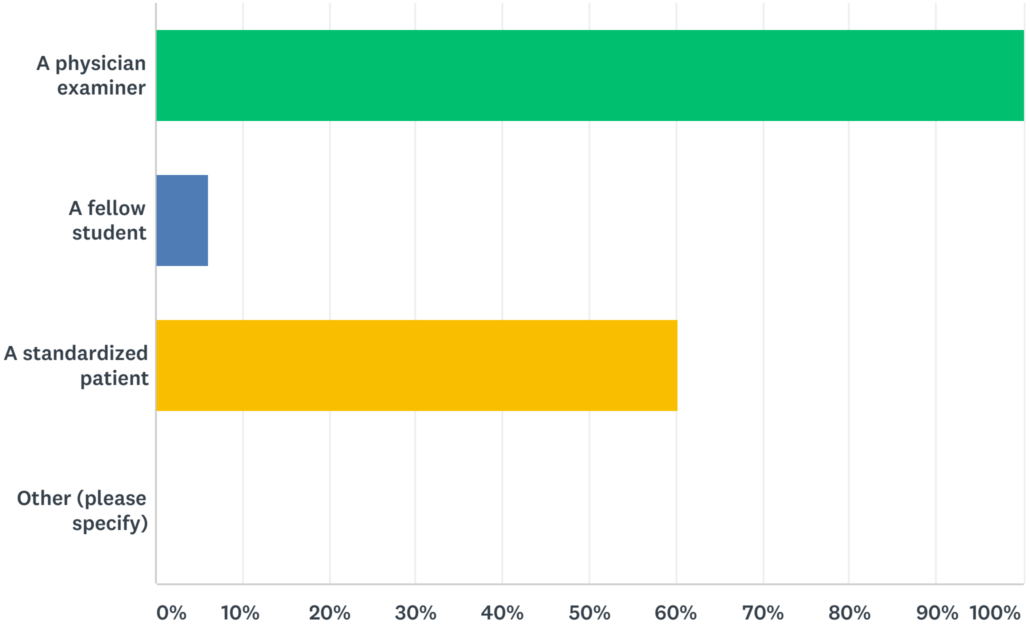

ANSWER CHOICES	RESPONSES	
A physician examiner	100.00%	98
A fellow student	6.12%	6
A standardized patient	60.20%	59
Other (please specify)	0.00%	0
Total Respondents: 98		

Q6 Related to content:

Answered: 96 Skipped: 62

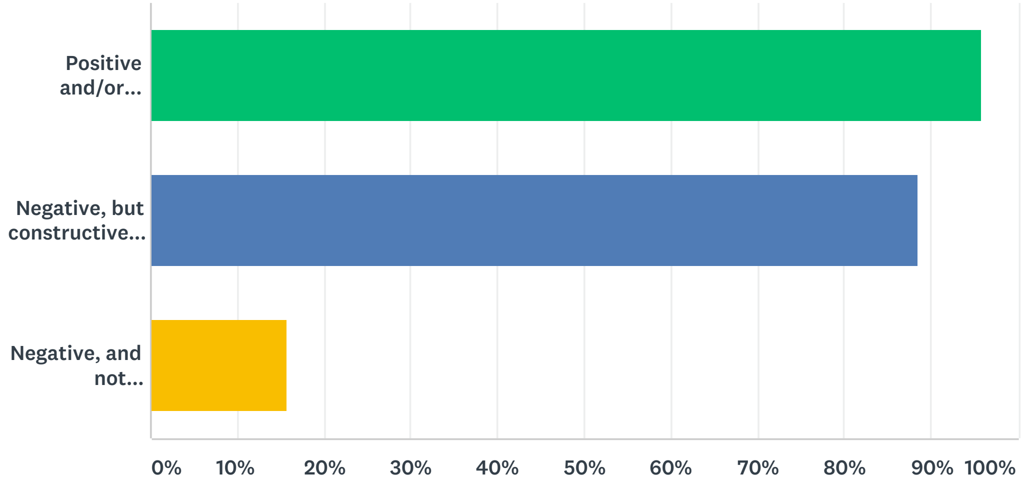

ANSWER CHOICES	RESPONSES	
Positive and/or reassuring (e.g., great job, you hit all the major points)	95.83%	92
Negative, but constructive (e.g., next time you should ensure that you ask about B symptoms)	88.54%	85
Negative, and not constructive (e.g., you really don't have a good approach to chest pain)	15.63%	15
Total Respondents: 96		

Q7 Related to rapport with patient:

Answered: 89 Skipped: 69

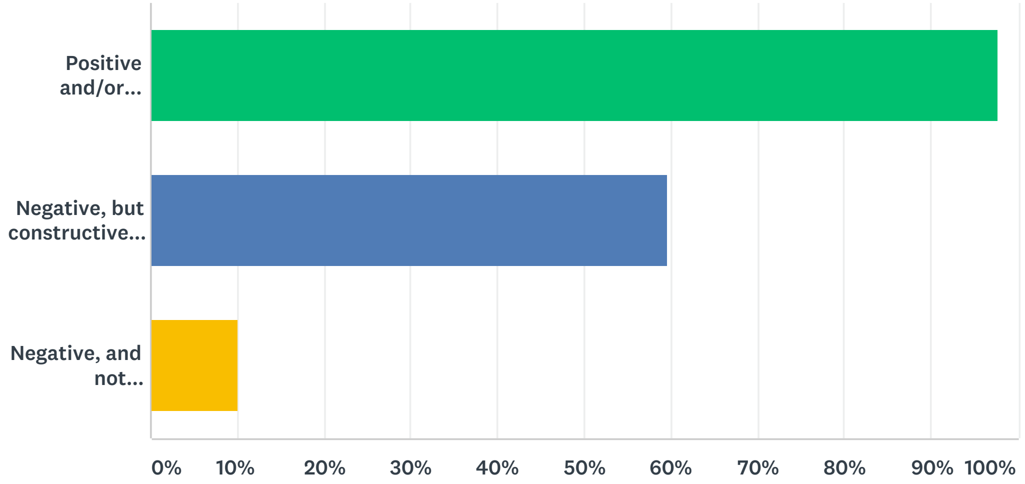

ANSWER CHOICES	RESPONSES	
Positive and/or reassuring (e.g., great job- you really listened to the patient's concerns)	97.75%	87
Negative, but constructive (e.g., next time you should ask more open-ended questions at the beginning of the interview)	59.55%	53
Negative, and not constructive (e.g., you really didn't make an effort to connect with the patient)	10.11%	9
Total Respondents: 89		

Q8 Related to self:

Answered: 83 Skipped: 75

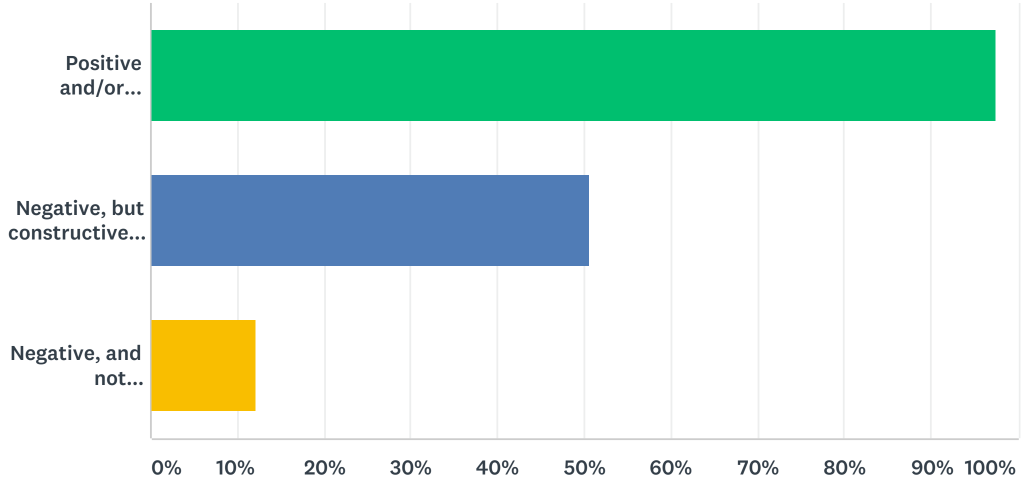

ANSWER CHOICES	RESPONSES	
Positive and/or reassuring (e.g., you have a really good bedside manner)	97.59%	81
Negative, but constructive (e.g., in the future, you should try to ensure you make statements to your patient demonstrating your understanding and compassion to their concerns)	50.60%	42
Negative, and not constructive (e.g., you really lacked empathy)	12.05%	10
Total Respondents: 83		

Q9 6. Have you ever had an emotional reaction (e.g., embarrassment, anger, etc.) to negative verbal or nonverbal (e.g., eye-rolling, negative tone of voice) feedback received during an OSCE that you deemed was too harsh, rude, unfair or inappropriate?

Answered: 97 Skipped: 61

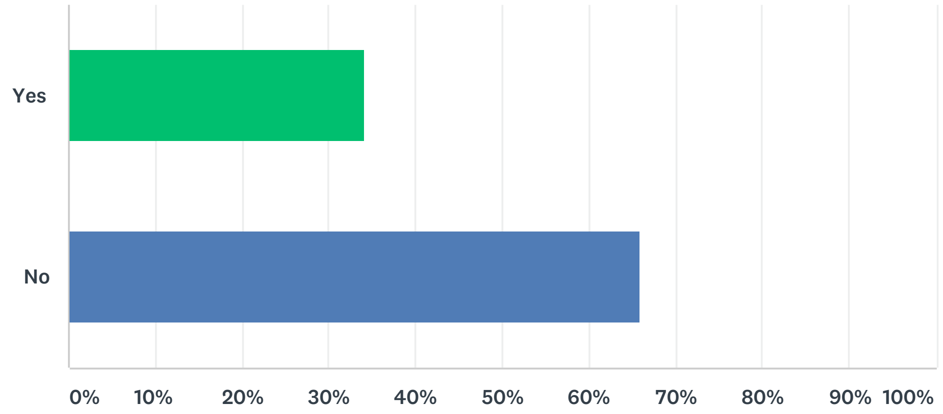

ANSWER CHOICES	RESPONSES	
Yes	34.02%	33
No	65.98%	64
TOTAL		97

Q10 7. What was your emotional reaction? Select as many as applicable.

Answered: 27 Skipped: 131

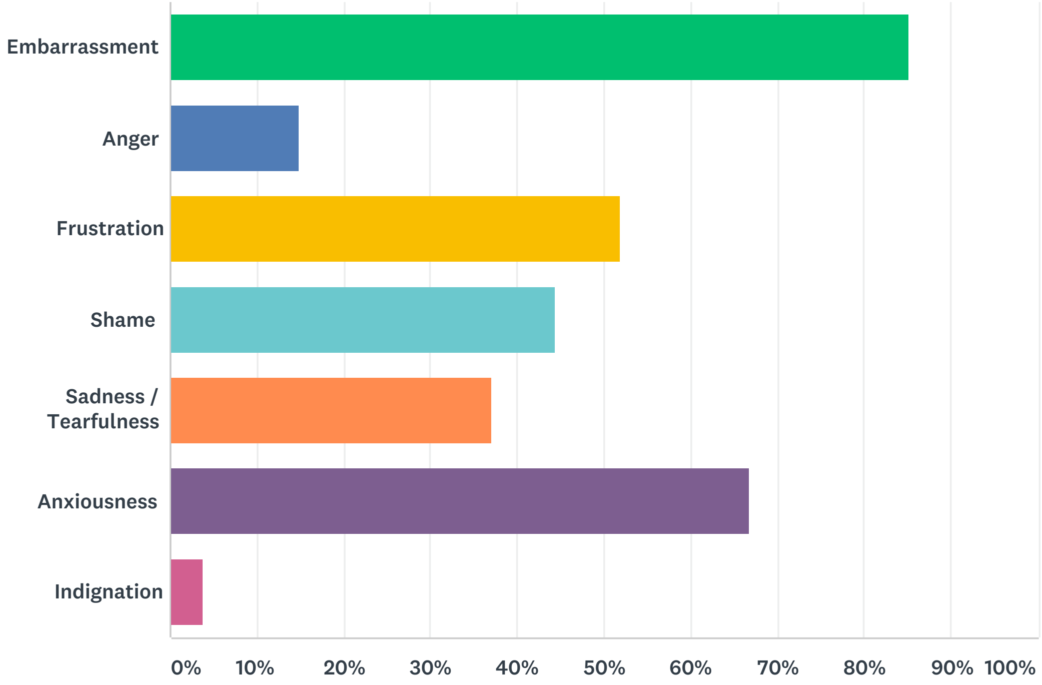

ANSWER CHOICES	RESPONSES	
Embarrassment	85.19%	23
Anger	14.81%	4
Frustration	51.85%	14
Shame	44.44%	12
Sadness / Tearfulness	37.04%	10
Anxiousness	66.67%	18
Indignation	3.70%	1
Total Respondents: 27		

Q11 8. Please provide examples of the verbal and/or nonverbal feedback that led to your emotional reaction.

Answered: 27 Skipped: 131

Q12 9. How did this affect your performance on subsequent stations during the OSCE?

Answered: 27 Skipped: 131

Q13 10. How did this feedback affect your preparation for and/or performance on subsequent OSCEs?

Answered: 27 Skipped: 131

Q11 8. Please provide examples of the verbal and/or nonverbal feedback that led to your emotional reaction.

Answered: 27 Skipped: 131

#	RESPONSES	DATE
1	eye rolling "looks like everyone in the city is going to get tetanus" - referring to the fact that several students forgot to ask about tetanus status at this station	6/18/2019 10:03 AM
2	patronizing	6/17/2019 6:35 PM
3	Facilitator picked on a specific word I used when evaluating my own experience (not something I said during patient interaction)	6/17/2019 2:17 PM
4	a	5/27/2019 8:54 AM
5	Examiner rolling their eyes at a response I gave, replying with uncertainty to something I said (e.g. saying okay sarcastically).	2/26/2019 10:26 AM
6	Examiner said I ignored patient comfort and that when the patient had said she was in pain I had just answered with "OK". I had actually asked the patient if she was OK to continue. Patient defended me to the examiner. Was frustrated because he accused me of doing something I had not done and it felt like he had not even been paying attention to my performance.	2/25/2019 12:32 AM
7	Pointing out things I had done wrong, especially very picky details (for example I used the term "authorities" rather than "police" and was criticized heavily for this)	2/24/2019 10:13 PM
8	Can't think of specifics.	2/23/2019 8:49 PM
9	I have had an examiner roll their eyes near the end of the station while answering questions related to the station.	2/22/2019 5:54 PM
10	Although his feedback was constructive (i.e. don’t forget to review the nerve root origins for reflexes). His tone of voice was was harsh, and expelled disappointment. When he went on to ask the nerve root questions on the lower limb reflexes he asked “Are you sure you want to continue?” In a patronizing tone which made me feel that I definitely was going to get the question wrong even though I knew the lower limb reflexes exceedingly better than upper limbs.	2/22/2019 4:26 PM
11	Rolling the eyes or saying what else? And keep probing	2/22/2019 1:54 PM
12	don't remember specifics	2/20/2019 12:29 AM
13	Very blunt negative feedback, continuing correction on technique which I felt I had never been taught and should not be expected to know, ongoing negative feedback not interspersed with reassurance, felt very "lecture-y"	2/4/2019 10:35 AM
14	Misinterpretation of my interaction/lack of empathy with a patient	2/3/2019 5:25 PM
15	Non-verbal feedback of disinterest and eye rolling when I did not know the answer to one of the PEP questions.	2/3/2019 5:12 PM
16	I was told that "you really didnt know what to do on this station eh?"	2/3/2019 2:07 PM
17	"Do you even know what you are saying?"	2/2/2019 11:14 AM
18	Stern tone, flat expression, repeatedly asking "anything else?" (so then I knew I was missing answers)	2/1/2019 6:32 PM
19	Mainly I’ve had challenges with non verbal communication such as eye rolling, loud sighing, etc.	2/1/2019 4:09 PM
20	During an OSCE station when questions were asked, I provided an incorrect answer, and the examiner obviously displayed his displeasure.	2/1/2019 12:47 PM
21	The examiner told me I didn’t do certain steps that I did complete. He suggested by not doing a step perfectly it was “unacceptable” and implied I was therefore inadequate	2/1/2019 12:46 PM
22	Facial expressions that showed that he was unimpressed	2/1/2019 12:46 PM

23	I was told I didn't listen to the patient, I disagreed	1/26/2019 2:42 PM
24	NA	1/24/2019 4:15 PM
25	"It was completely wrong"	11/24/2018 2:33 PM
26	I had a very difficult patient interaction with a standardized patient in my second year. The context of the OSCE station was to talk to a young woman about her sexual assault (Obs/Gyn station). The issue was that I didn't actually ask directly about sexual assault in my history with the patient, and the patient (to be honest) was not acting in any way that would make me think that she was sexually assaulted or that that it would be appropriate to ask. At the end of the station, the physician preceptor told me that I am not very empathetic, not attentive, and that I missed a large piece of that station. I was very offended and I almost burst into tears because I definitely consider myself an empathetic, attentive, and kind person. This interaction really affected me.	11/20/2018 3:06 PM
27	You looked at your paper and wrote too much down. You didn't have a good approach.	11/12/2018 11:26 AM

Q12 9. How did this affect your performance on subsequent stations during the OSCE?

Answered: 27 Skipped: 131

#	RESPONSES	DATE
1	difficulty concentrating	6/18/2019 10:03 AM
2	made me better	6/17/2019 6:35 PM
3	Negatively. Made me less sure of myself	6/17/2019 2:17 PM
4	a	5/27/2019 8:54 AM
5	It was rattling to have that happen in the station and I try my best not to take that into the next one, but there has been a time where I went into the next stations very anxious and performed poorly.	2/26/2019 10:26 AM
6	it did not affect my performance I pushed it to the back of my mind and moved on.	2/25/2019 12:32 AM
7	It increased my anxiety levels and decreased my confidence going forward, especially if I missed some obvious points on the station	2/24/2019 10:13 PM
8	It didn't	2/23/2019 8:49 PM
9	I felt more insecure and less confident.	2/22/2019 5:54 PM
10	I was so rattled I ended up forgetting my stethoscope in the examination room. This resulted in the subsequent cardio related station very difficult, as I had to run back to the room to grab it before I could continue.	2/22/2019 4:26 PM
11	Did not really affect my performance	2/22/2019 1:54 PM
12	I thought about previous feedback in subsequent stations which I feel negatively impacted by performance	2/20/2019 12:29 AM
13	I felt a bit flustered going forwards	2/4/2019 10:35 AM
14	Fortunately, it was my last station but I was really nervous for future OSCE	2/3/2019 5:25 PM
15	I felt upset and that I had already failed going into my next stations.	2/3/2019 5:12 PM
16	i was way more anxious walking into future stations. I had lost alot of my confidence for the rest of the exam.	2/3/2019 2:07 PM
17	Heightened level of anxiety for remaining stations.	2/2/2019 11:14 AM
18	Increased nervousness	2/1/2019 6:32 PM
19	It made me extremely anxious and negatively impacted my performance on future stations.	2/1/2019 4:09 PM
20	Thought about it during the subsequent station, and it may have impacted my performance.	2/1/2019 12:47 PM
21	This was feedback right at the end but it wouldn't have negatively impacted me if I had later stations	2/1/2019 12:46 PM
22	Made me more anxious, couldn't think clearly	2/1/2019 12:46 PM
23	Negatively affected my performance.	1/26/2019 2:42 PM
24	slightly hindered	1/24/2019 4:15 PM
25	Completely through me "off my game"	11/24/2018 2:33 PM
26	It made the next station more difficult, for sure, since I was almost in tears and I found it difficult to recompose myself. The next station was a little bit more objective (I think that it was a physical exam) so I just put my feelings aside and moved on	11/20/2018 3:06 PM
27	Was self-conscious for subsequent stations and more anxious, clouding my thinking	11/12/2018 11:26 AM

schools that sent out:
uOttawa
Western
Queens
Toronto

Q13 10. How did this feedback affect your preparation for and/or performance on subsequent OSCEs?

Answered: 27 Skipped: 131

#	RESPONSES	DATE
1	increased pretest anxiety	6/18/2019 10:03 AM
2	studied harder to screw over these type of assholes	6/17/2019 6:35 PM
3	More anxiety.	6/17/2019 2:17 PM
4	a	5/27/2019 8:54 AM
5	I was still able to do okay overall, on my OSCE in this instance. I didn't feel more anxious going into subsequent OSCEs.	2/26/2019 10:26 AM
6	Have not had a subsequent osce yet.	2/25/2019 12:32 AM
7	It made me want to prepare more, not only for the sake of passing the stations, but also for avoiding feelings of embarrassment.	2/24/2019 10:13 PM
8	Made me realize how to approach further OSCEs	2/23/2019 8:49 PM
9	It made me more nervous for future OSCEs, especially the idea of having that examiner again, but I dont think it helped me to prepare any better.	2/22/2019 5:54 PM
10	I made sure to prepare extensively for future follow-up questions for future OSCEs so that I would not feel emabarrassed again.	2/22/2019 4:26 PM
11	N/A	2/22/2019 1:54 PM
12	It made me more nervous to do future OSCEs	2/20/2019 12:29 AM
13	I don't think it affected my performance.	2/4/2019 10:35 AM
14	It was a misinterpretation of the physician regarding my performance but I practiced more.	2/3/2019 5:25 PM
15	Made me prepare more but also worry more.	2/3/2019 5:12 PM
16	My next OSCE, i studied for, for 4 times as much as much as I would usually prepare for an OSCE	2/3/2019 2:07 PM
17	I have not had a subsequent OSCE.	2/2/2019 11:14 AM
18	It didn't	2/1/2019 6:32 PM
19	I think it made me more nervous than necessary for future OSCEs which reduced my studying efficacy.	2/1/2019 4:09 PM
20	It didn't really.	2/1/2019 12:47 PM
21	It made me more stressed and self consy	2/1/2019 12:46 PM
22	It didn't	2/1/2019 12:46 PM
23	Did not.	1/26/2019 2:42 PM
24	NA	1/24/2019 4:15 PM
25	Made me extremely nervous and knocked my sense of self confidence	11/24/2018 2:33 PM
26	I think that it made me keep in mind that I need to really interact with my SPs in a sensitive way, and that I shouldn't be shy to ask sensitive questions and to ask what is really on my mind.	11/20/2018 3:06 PM
27	Tried to prepare more but can never prepare enough. Feeding during the OSCE is stressful. All I want to do is finish a station, have a moment to clear my head before moving on to the next station, and not having to absorb feedback. It would be helpful to review at the end, but not during. There is very little time to finish, get out of the room, especially when the rooms aren't close together. I would really benefit from 30 seconds to clear my head.	11/12/2018 11:26 AM
