# Supplementary material for: Potential of feedback during objective structured clinical examination to evoke an emotional response in medical students in Canada
Source: J Educ Eval Health Prof. 2020 Feb 18;17:5. doi: 10.3352/jeehp.2020.17.5 (PMC7136617; doi:10.3352/jeehp.2020.17.5)
Supplement: Supplementary file 2 — Supplement 1. Survey questionnaire to explore the feedback received by medical students during OSCEs in the University of Ottawa Faculty of Medicine from November 2018 to June 2019. [file jeehp-17-05-suppl.pdf]

This study is being done to explore the feedback received by medical students during OSCEs. Feedback received during an OSCE is often perceived as useful in promoting learning, but it may also lead to powerful emotional reactions that can potentially impact performance on subsequent stations. We hope that this study will help us to gain insights into how students perceive the feedback they receive during OSCEs.

Participation is completely voluntary. If you decide to participate, you are free to discontinue responding to the survey at any time, without penalty or loss. Survey responses are anonymous and no names or contact information will be collected ; therefore, once the completed survey has been submitted, withdrawal will not be possible.

Start Survey ◯

1. In what year of medical school are you currently enrolled?

   - 2nd
   - 3rd
   - 4th
   - Other ___________________

2. How many OSCEs have you participated in as a medical student?
   - 0
   - 1
   - 2
   - 3
   - > 3

3. During your OSCEs, have you ever received verbal feedback from an examiner?
   - Yes (if yes, jump to question 4)
   - No (if no, end survey)

4. Who provided the verbal feedback? Select as many as applicable
    - A physician examiner
    - A fellow student
    - A standardized patient
    - Other_____________

5. How would you characterize the type of verbal feedback you received? Select ask many as applicable

    Related to content:

    - Positive and/or reassuring (e.g., great job, you hit all the major points)
    - Negative, but constructive (e.g., next time, you should ensure that you ask about B symptoms)
    - Negative, and not constructive (e.g., you really don't have a good approach to chest pain)

    Related to rapport with patient:

    - Positive and/or reassuring (e.g., great job - you really listened to the patient's concerns)
    - Negative, but constructive (e.g., next time you should ask more open-ended questions at the beginning of the interview)
    - Negative, and not constructive (e.g., you really didn't make an effort to connect with the patient)

    Related to self:

    - Positive and/or reassuring (e.g., you have a really good bedside manner)
    - Negative, but constructive (e.g., in the future, you should try to ensure you make statements to your patient demonstrating your understanding and compassion to their concerns.)
    - Negative, and not constructive (e.g., you really lacked empathy)

6. Have you ever had an emotional reaction (e.g., embarrassment, anger, etc.) to negative verbal feedback or nonverbal (e.g., eye-rolling, negative tone of voice) received during an OSCE that you deemed was "too harsh", rude, unfair or inappropriate?
   - Yes (if yes, jump to next question)
   - No (if no, end of survey)

7. What was your emotional reaction? Select as many as applicable
   - Embarrassment
   - Anger
   - Frustration
   - Shame
   - Sadness/Tearfulness
   - Anxiousness
   - Indignation
   - Other ________________

8. Please provide examples of the verbal and/or nonverbal feedback that led to your emotional reaction

   ___________________________________(FREE TEXT)

9. How did this feedback affect your performance on subsequent stations during the OSCE?

   ____________________________________(FREE TEXT)

10. How did this feedback affect your preparation for and/or performance on subsequent OSCE exams?

_______________________________________(FREE TEXT)

Thank you for completing our survey!
